# Supplementary material for: Hip Fracture Prevention in Osteoporotic Elderly and Cancer Patients: An On-Line French Survey Evaluating Current Needs
Source: Medicina (Kaunas). 2020 Aug 7;56(8):397. doi: 10.3390/medicina56080397 (PMC7466279; doi:10.3390/medicina56080397)
Supplement: Supplementary file 1 [file medicina-56-00397-s001.pdf]

medicina

MDPI

Supplementary material

Elicitation questionnaire

**Section I = introduction (about the survey and the studied device)**	
**Do you know the medical device Y-STRUT®?**	YES, already used/prescribed<br>YES, but non-used/prescribed<br>NO, never heard about it
How did you know about this questionnaire?	Scientific society, federation or study group<br>Journal, newspaper or newsletter<br>Direct contact by the PhD student<br>Other: specify
Do you want more information about this product and/or the results of this survey?	YES / NO
Have you already answered this survey, even incompletely?<br>(this question enables to avoid duplicates)	YES / NO
Section II = about the practitioner	
You are?	A man / A woman
What is your age?	Less than 35 years old<br>35-45 years old<br>45-55 years old<br>More than 55 years old
Professional experience?	Less than 10 years<br>10-20 years<br>20-30 years<br>More than 30 years
What is your workplace in Hospital? *	University Hospital Centre (CHU)<br>Regional University Hospital Centre (CHRU)<br>Hospital Centre (CH)<br>Private
Where do you work? (French department)	Selection of a French department
What is your specialty?	Orthopedic surgeon<br>Rheumatologist<br>Geriatrician<br>Interventional radiologist<br>Oncologist<br>Radiotherapist<br>Other: specify
In which indication(s) are you likely to be interested in this device? *	Orthopedics-traumatology indication<br>Oncological indication
Section IIIA = Orthopedics-traumatology indication	
In relation to the studied device, will you be?	User / Prescriber / Both
How many patients with a hip fracture due to osteoporosis do you see per month in average?	< 5 / 5-12 / 12-20 / > 20 / NK
Estimated annual total?	< 10 / 10-20 / 20-50 / 50-100 / 100-200 / 200-300 / 300-500 / > 500 / Unknown
Which type of hip fracture do you see?	Mostly per trochanteric fractures<br>Mostly femoral neck fractures<br>Both equally<br>NK
What is the cause of their hip fracture?	Mostly a simple fall from its height<br>Mostly a fall from bed<br>Mostly a fall in the stairs<br>A little bit of everything<br>Other: specify<br>NK
What is the history of fractures of these patients? *	1 vertebra / Several vertebras / Wrist / Other / NK
How many contralateral hip fractures do you see per year in average?	0-5 / 5-10 / 10-20 / 20-50 / > 50 / NK
Your patients suffering for hip fracture(s) are?	Mainly women<br>Mainly men<br>Both equally
How old are these patients in majority?	Less than 65 years old

	65-75 years old<br>75-85 years old<br>More than 85 years old
Where do these patients live BEFORE contralateral hip fracture?	Mostly at home<br>Mostly in a health care facility<br>Both equally<br>NK
Where do these patients live AFTER contralateral hip fracture?	Mostly at home<br>Mostly in a health care facility<br>Both equally<br>NK
Osteoporosis diagnosed before fracture?	Mostly YES / Mostly NO / Both equally / NK
Medical treatment for osteoporosis before the first hip fracture?	YES / NO / NK
Which medical treatment is prescribed in majority?	Bisphosphonates / Calcium-vitamin D / Estrogen / Denosumab / THS / Teriparatide / PTH / strontium ranelate / other: specify
Which fixation material do you use for hip fracture treatment?	Nail<br>Screw and plate<br>Both<br>Other: specify<br>I do not operate
What are the main criteria of material choice for hip fracture treatment? *	Fracture type<br>Pain<br>Stability, rehabilitation<br>Easy-to-use<br>Material availability<br>Cost<br>NK
Which anesthesia is the most used for hip fracture treatment?	Mostly general anesthesia<br>Mostly spinal anesthesia<br>Both similarly<br>Other: specify<br>NK
What is the mean surgical time (without complications) for hip fracture treatment?	< 30min / 30-60 min / > 1h / > 2h / NK
Do you prescribe a medical treatment for osteoporosis after the first hip fracture?	Mostly YES<br>Mostly NO<br>I am not a prescriber
What is the mean life expectancy for patients after the FIRST hip fracture with the usual medical/surgical treatment?	< 1 month<br>< 6 months<br>< 12 months<br>> 12 months<br>NK
How many deaths per year do you estimate at 1 year after the FIRST hip fracture with the usual medical/surgical treatment?	0-5 / 5-10 / 10-15 / 15-20 / > 20 / NK
What is the mean life expectancy for patients after the SECOND hip fracture (contralateral) with the usual medical/surgical treatment?	< 1 month<br>< 6 months<br>< 12 months<br>> 12 months<br>NK
How many deaths per year do you estimate at 1 year after the SECOND hip fracture with the usual medical/surgical treatment?	0-5 / 5-10 / 10-15 / 15-20 / > 20 / NK
What reduction in the risk of contralateral fracture at 1 year would lead you to use this new implantable medical device in prevention?	30% / 50% / 70% / 90% / NK
Which benefit(s) would lead you to use/prescribe this new device? *	Refracture rate at 1 year<br>Life expectancy at 1 year<br>Quality of life<br>Comorbidities<br>Patient's dependency<br>I do not see benefit, I will not use/prescribe it
Which risk(s) would lead you to not use/prescribe this new device? *	Surgical risk<br>Not enough clinical experience<br>I think that the benefits-to-risks ratio is unfavorable<br>Other: specify<br>I do not see major risk, this device is a good solution

What would be your prevention strategy of the contralateral hip with this new implantable medical device?	Simultaneously with the 1st fracture treatment<br>Postponed<br>No prevention<br>NK
Post-poned prevention strategy?	Before hospital exit<br>At the 1-month visit<br>At the 3-month visit<br>After 6 months
Will you be willing to use/prescribe this new implantable medical device in prevention?	YES / NO / NK
Are you satisfied by the questionnaire?	(scale) 100: absolutely, 0: not satisfied at all
Section IIIB = Oncology indication	
In relation to the studied device, will you be?	User / Prescriber / Both
How many patients with metastatic pre-fractural lesions at the proximal femur do you see per month in average?	< 5 / 5-12 / 12-20 / > 20 / NK
Estimated annual total?	< 10 / 10-20 / 20-50 / 50-100 / 100-200 / 200-300 / 300-500 / > 500 / NK
Which type of lesions do you see at the proximal femur in majority?	Lesion at the femoral head<br>Lesion at the femoral neck<br>Per trochanteric lesion<br>Lesion at the diaphysis<br>All locations equally<br>NK
How many hip fractures linked to these lesions do you see per year in average?	< 10 / 10-20 / 20-50 / 50-100 / 100-200 / 200-300 / 300-500 / > 500 / NK
What is the mean life expectancy for patients after a hip fracture with the usual medical/surgical treatment?	< 1 month<br>< 6 months<br>< 12 months<br>> 12 months<br>NK
How many deaths per year do you estimate at 1 year after a hip fracture with the usual medical/surgical treatment?	0-5 / 5-10 / 10-15 / 15-20 / > 20 / NK
In accordance to the ECOG score, how do you classify the patients suffering from lesions at the proximal femur?	1: Mostly fully active<br>2: Mostly restrained in physical activities, but outpatient, professional activity or at home ok<br>3: Mostly outpatient, autonomous but without professional activities or at home<br>4: Mostly poor, assisted/bedridden more than 50% of the day<br>5: Mostly disabled, permanently assisted/ bedridden, with assistance<br>All proposals equally<br>NK
Medical treatment for osteolytic metastasis pre-fractural lesions at the proximal femur?	YES / NO / NK
Which medical treatment is prescribed in majority?	Chemotherapy<br>Local radiotherapy<br>Chemotherapy + local radiotherapy<br>Other: specify
Which local treatment is often associated? *	Embolization<br>Ablation (radiofrequency or cryotherapy)<br>Radiotherapy<br>Other: specify<br>No treatment<br>NK
Which type of primary cancer is associated to osteolytic metastasis at the proximal femur in majority? *	Lung / Breast / Prostate / Kidney / Melanoma<br>Other: specify / NK
Do you propose to these patients a preventive treatment for proximal femur fracture?	Mainly NO<br>Mainly YES, addressed to the orthopedics service<br>Mainly YES, addressed to a multidisciplinary meeting<br>Other: specify
What is the main decision criterium for proximal femur fracture prevention?	Mirels' score / Pain / ECOG / Other: specify / NK
About the Mirels' score1	Choice between 3 and 12
About the pain	Choice between 1 and 10
About the ECOG score	Choice between 1 and 5

Which preventive treatment do you propose, in majority, against proximal femur fracture?	Osteosynthesis in the orthopedics service<br>Screwing + cementoplasty<br>Cementoplasty alone<br>I do not do prevention<br>Other: specify
What is(are) the main criterium(a) of choice for proximal hip fracture prevention?	Size of the lesion<br>Pain<br>Easy-to-use<br>Material availability<br>Cost<br>NK
Which anesthesia is the most used for proximal hip fracture prevention?	Mostly general anesthesia<br>Mostly spinal anesthesia<br>Both similarly<br>Other: specify<br>NK
Where is done the preventive treatment in majority?	In the orthopedics operative room (OR)<br>In the interventional radiology OR
What is the mean surgical time (without complications) for proximal hip fracture prevention?	< 30min / 30-60 min / > 1h / > 2h / NK
Which benefit(s) would lead you to use/prescribe this new device? *	Refracture rate at 1 year<br>Life expectancy at 1 year<br>Quality of life<br>Comorbidities<br>Patient's dependency<br>I do not see benefit; I will not use/prescribe it
Which risk(s) would lead you to use/prescribe this new device? *	Surgical risk<br>Not enough clinical experience<br>I think that the benefits-to-risks ratio is unfavorable<br>Other: specify<br>I do not see major risk; this device is a good solution
Will you be willing to use/prescribe this new implantable medical device in prevention?	YES / NO / NK
Are you satisfied by the questionnaire?	(scale) 100: absolutely, 0: not satisfied at all

* several responses possible; NK=unknown; [1] Mirels classification- Metastatic disease in long bones and impending pathologic fracture - Jawad et al. 2010 [1]

1. Jawad, M.U.; Scully S.P. In brief : Classifications in brief: Mirels' classification: Metastatic disease in long bones and impending pathologic fracture. *Clin. Orthop. Relat. Res*. **2010**, 468, 2825–2827.
